# Polypus of the Rectum—Clinical Lecture

**Published:** 1847-01

**Authors:** 


*Polypus of the Rectum—Clinical Lecture, by M. Guersant, Jun.*—A little girl was lately admitted into the Hôpital des Enfans under the following circumstances:—Two months since, blood was passed in small quantities with the motions, and during the expulsion of the fæces a red tumour was observed to protrude from the anus. When a child presenting these symptoms is brought to a surgeon, it is natural to suppose that the case is one of prolapsus ani: this opinion must not, however, be exclusively adopted; we have several times detected in such children the presence of polypus in the rectum. It is worthy of remark, that no ancient or modern work on the diseases of infancy has alluded to this form of disease. M. Stoltz, in 1831, was the first to give a history of the symptoms, and two cases only were previously on record: one published in "Hobold's Journal," 1828, by M. Schneider; the other, by Dr. Lange, of Berlin, in 1776.

The first signs are those above mentioned, viz., sanguineous motions, and tenesmus; defæcation gradually becomes more and more impeded, in proportion as the polypus increases in size, and is accompanied by violent efforts of expulsion, which force out from the intestine a red, even tumour, at first easily reduced. We have often noticed a sign which we conceive to be of some value—it is the presence of a groove or furrow on the surface of the fæces, caused by the presence of the polypus; but the issue of the tumour through the anus is the only certain diagnostic sign. During the first period of the complaint the swelling is round, and slightly flattened at its sides; the external segment is more voluminous than its intestinal portion.

Authors do not agree upon the nature of these growths: some consider them to be of a fibro-cellular structure; others, on the contrary, believe them to be always of a mucous texture. Thus, M. Stoltz thinks, that in many cases, they are the result of frequently repeated prolapsus ani, a portion of mucous membrane incarcerated in the ring of the sphincters becoming congested, swollen, and pediculated after a certain period. Such may be, in some instances, the real mode according to which polypus of the rectum is generated, but there are many exceptions—thus, polypus is frequently observed on mucous surfaces unprovided with sphincter muscles; besides, prolapsus ani has other well-known results; let us add, that polypus has been observed in subjects who had never suffered from prolapsus ani. We have usually found these polypi to consist of a mucous sheath borrowed from the mucous membrane of the rectum, enveloping a spongy texture.

So long as the tumours do not issue through the rectum, a hemorrhagic discharge from the intestine, and the nature of the stains of the linen, cannot furnish a positive basis to the diagnosis; the finger must be introduced *per anum*, the pedicle of the tumour accurately circumscribed, and the spot of its insertion precisely ascertained. Although the prognosis must usually be favourable, still the abundance and frequent return of hemorrhage may seriously injure a child's health, and it is therefore necessary to come to a speedy determination when once the nature of the disease has been correctly ascertained. The spontaneous cure can be readily understood; the polypus, gradually expelled by efforts of defæcation, drags more and more upon the pedicle, which daily diminishes in diameter and increases in length, until at last it yields to the frequent repetitions of the efforts; the polypus falls away, and a spontaneous removal of the complaint may be said to have taken place. Perhaps even such polypi may have been the unknown and unsuspecting cause of rebellious diarrhœa, which ceased after their spontaneous expulsion, after medicines of various kinds had previously been exhibited without success.

We do not approve of the use of caustics, because their action is uncertain, and their application in cavities like the rectum is always inconvenient, and sometimes unsafe. Excision we adopt only when the neck of the tumour is very narrow, because even a slight hemorrhage may endanger the life of a child. We prefer to all other methods, simple ligature, because all danger from loss of blood is obviated, and the little patients suffer no pain: in general, when the implantation of the pedicle is not very high, the threads may be simply carried with the finger round the neck; but, in the contrary case, the introduction of the speculum ani considerably facilitates the operation.—*London Medical Times.*

